# Attention is Required for Maintenance of Feature Binding in Visual Working Memory

**DOI:** 10.1080/17470218.2013.852232

**Published:** 2014-06-01

**Authors:** Nahid Zokaei, Maike Heider, Masud Husain

**Affiliations:** 1 Department of Experimental Psychology, University of Oxford, Oxford, UK; 2 Nuffield Department of Clinical Neurosciences, University of Oxford, Oxford, UK

**Keywords:** Visual working memory, Attention, Executive resources, Binding failures

## Abstract

Working memory and attention are intimately connected. However, understanding the relationship between the two is challenging. Currently, there is an important controversy about whether objects in working memory are maintained automatically or require resources that are also deployed for visual or auditory attention. Here we investigated the effects of loading attention resources on precision of visual working memory, specifically on correct maintenance of feature-bound objects, using a dual-task paradigm. Participants were presented with a memory array and were asked to remember either direction of motion of random dot kinematograms of different colour, or orientation of coloured bars. During the maintenance period, they performed a secondary visual or auditory task, with varying levels of load. Following a retention period, they adjusted a coloured probe to match either the motion direction or orientation of stimuli with the same colour in the memory array. This allowed us to examine the effects of an attention-demanding task performed during maintenance on precision of recall on the concurrent working memory task. Systematic increase in attention load during maintenance resulted in a significant decrease in overall working memory performance. Changes in overall performance were specifically accompanied by an increase in feature misbinding errors: erroneous reporting of nontarget motion or orientation. Thus in trials where attention resources were taxed, participants were more likely to respond with nontarget values rather than simply making random responses. Our findings suggest that resources used during attention-demanding visual or auditory tasks also contribute to maintaining feature-bound representations in visual working memory—but not necessarily other aspects of working memory.

What do working memory and attention systems share? While many authors have pointed out the intimate connections between these two processes—in terms of both behaviour and neural substrate (e.g., Awh & Jonides, 2001; Chun, 2011; Chun, Golomb, & Turk-Browne, 2011; Lavie, 2005; Rensink, 2000; Wheeler & Treisman, 2002)—it remains unclear how much overlap there is between them. Some go as far as defining visual working memory as active maintenance of attention to visual information (Chun, 2011) highlighting the very close relationship between these processes. Indeed, several studies have shown that spatial working memory load interferes with concurrently performed visual search tasks (e.g., Emrich, Al-Aidroos, Pratt, & Ferber, 2010; Oh & Kim, 2004; Woodman & Luck, 2004). Recently, an important controversy has arisen about whether attention is required for the maintenance of feature-bound objects in working memory (Baddeley, Allen, & Hitch, 2011; Chun et al., 2011; Fougnie & Marois, 2009; Luck & Vogel, 1997). Here, we investigate this issue using a relatively new technique that allows us to examine the precision of recall and decompose the types of error made when participants retrieve an item from working memory (Bays, Catalao, & Husain, 2009; Zhang & Luck, 2008).

A central quality of working memory is its limited capacity (Cowan, 2001), a property shared by attention processes (Chun et al., 2011). Cowan has argued for a capacity-limited system for maintenance of information available for conscious awareness, with a limit of about four chunks of information in healthy adults (Cowan, 1998, 2001, 2005). In the visual domain, the chunks are expressed as the number of integrated objects—that is, with all features that belong to an object correctly bound together. In fact, in line with this framework, object-based theories of visual working memory propose that there are a limited number of discrete memory slots, each storing or maintaining information regarding an individual object consisting of different features (Anderson, Vogel, & Awh, 2011; Luck & Vogel, 1997; Luria & Vogel, 2011).

However, within object-based theories of working memory, the role of attention has yet to be established. One line of research has argued that maintenance of bound features in working memory is automatic and demands the same amount of resources as maintaining individual features (Luck & Vogel, 1997; Vogel, Woodman, & Luck, 2001). In a series of pioneering experiments, Luck and Vogel (1997) reported that working memory performance was unaffected by the number of features in the objects to be remembered. Therefore, according to these object-based models of working memory, although initial feature binding relies on attention, maintenance of stored items can be achieved in the absence of attention resources.

Support for such a proposal comes also from studies that employ working memory and attention tasks concurrently. The rationale behind such dual-task studies is to demonstrate overlap in cognitive processes. For example, if memory for binding depends more on attention resources, loading these resources should result in larger impairments in memory in trials where information was stored in a bound form than for “disintegrated” features. Some previous studies have reported similar levels of impairment in memory for independent features and feature-bound objects using dual working memory/attention tasks (Allen, Baddeley, & Hitch, 2006; Allen, Hitch, Mate, & Baddeley, 2012; Baddeley et al., 2011; Johnson, Hollingworth, & Luck, 2008; Luck & Vogel, 1997; Yeh, Yang, & Chiu, 2005; see also Delvenne, Cleeremans, & Laloyaux, 2010; Gajewski & Brockmole, 2006). However, it has recently been argued that the extent to which automatic feature binding occurs in working memory depends on whether the feature-bound objects are perceived as a coherent object (Ecker, Maybery, & Zimmer, 2012). These findings would therefore be consistent with automatic maintenance of bound features in working memory, at least under some conditions.

Contrary to these claims, other researchers have argued for a role of attention in working memory maintenance, emphasizing that in situations where attention is withdrawn, object representations collapse into disintegrated features in working memory (Rensink, 2000; Wheeler & Treisman, 2002). Wheeler and Treisman (2002) proposed a two-stage model of working memory: Within each feature dimension, working memory is limited to a few items but binding is maintained only by relying on attention processes. This model was based on the results of a study where participants were asked to detect either a change among individual features or a change in feature conjunction (binding condition) following a retention period. Crucially, they found that change-detection performance was impaired in trials where participants were asked to detect a change in the binding condition compared to changes in individual or multiple features (Wheeler & Treisman, 2002). The authors argued that since maintenance of integrated objects is more attentionally demanding, more errors in performance arise under the binding condition. Note that these findings were observed only when the whole memory array was presented at probe. Thus the significance of this effect is highly dependent on the methodology employed.

More recently, Chun and colleagues have put forward a taxonomy for attention, arguing that attention can be directed to internal representations—in the absence of sensory information—to maintain feature binding in integrated objects (Chun, 2011; Chun et al., 2011). According to this view, maintenance of bound features within a limited set of objects is dependent on “internal attention” resources. Consistent with this proposal, some studies using dual-task designs have shown large decrements in memory performance for integrated objects, compared to disintegrated features (Brown & Brockmole, 2010; Fougnie & Marois, 2009; Stefurak & Boynton, 1986).

As a result of these contradictory findings in the literature, there still remains a lack of consensus on the role of attention in maintenance of bound objects, despite its highlighted role in many theories of working memory (Chun, 2011; Wheeler & Treisman, 2002). One reason for divergent results might lie in the methodology used. In dual-task studies, attentional resources have so far been taxed using different tasks in the auditory or visual domains—performed either throughout the working memory task (i.e., at encoding, maintenance, and response phases) or at a specific phase (i.e., during maintenance and response phases only; e.g., Allen et al., 2012; Brown & Brockmole, 2010; Fougnie & Marois, 2009; Johnson et al., 2008). Further, until relatively recently, working memory performance has been measured using the change-detection paradigm, with comparisons made between performance across different conditions (e.g., memory for features vs. bound objects). Although change detection has been instrumental in advancing our understanding of working memory, limitations in this design may have resulted in the discrepancies observed in the literature. Recently, it has been shown that the magnitude of change critically influences change-detection performance (Keshvari, van den Berg, & Ma, 2012). Importantly, therefore, small changes can go undetected, particularly with increasing set sizes.

In studies that have focused on the issue of maintenance of bound objects, however, the magnitude of change has been set at an arbitrary value. Moreover, this value is different across different features. This is most vividly demonstrated by varied performance across features: Change detection is better for some features than for others (e.g., Allen et al., 2012; Wheeler & Treisman, 2002). Thus, interpreting performance in a binding condition, where one of two features of an object can change in any given trial, becomes problematic if the change is not equated across feature dimensions. Performance in the binding condition not only is subject to averaging across varied baselines but crucially also depends on the magnitude of change set for each feature in each experiment (Keshvari et al., 2012).

In addition, change-detection performance is influenced by probe presentation: Whole probe displays result in an decrease in accuracy in the binding condition, while single probe displays do not (Wheeler & Treisman, 2002). These limitations of change-detection design, together with differences in methodology and analysis between reported studies, can lead to discrepancies in findings (Allen et al., 2012). Thus, in order to understand the relationship between attention and feature binding in working memory, it is essential to use a task that allows us to investigate both feature only and feature-binding components of working memory without any changes in task difficulty confounding interpretation.

Tasks that measure the fidelity of working memory using adjustment techniques can potentially provide a more sensitive measure than change-detection paradigms (Bays & Husain, 2008; Wilken & Ma, 2004; Zhang & Luck, 2008). In adjustment tasks, participants are asked to reproduce the exact qualities of a stored feature. Using this methodology, it is possible to measure the fidelity or precision of memory on a continuous, analogue scale—rather than depend on binary change or no-change report. Crucially, instead of examining memory performance under tasks of varied difficulty (memory for features or integrated objects), we kept the working memory task constant and instead systematically manipulated the demand of the attention task during the maintenance period using a method previously shown to successfully load attentional resources (e.g., Forster & Lavie, 2008; Lavie, 2005; Lavie, Lin, Zokaei, & Thoma, 2009). Moreover, by applying a recent analytical technique (Bays et al., 2009; Zhang & Luck, 2008) we can distinguish different sources of errors that are systematically modulated by the demand of the attention task. This allows us to directly test whether altering attention demands affects feature binding of objects already maintained in working memory or alternatively influences the number of guesses (random responses) or resolution of feature representations.

In the present study, we examined the effects of deploying increasing levels of attention in a visual search task in maintenance of two objects defined by motion and colour (Experiment 1) and orientation and colour (Experiment 2), and for high levels of visual search difficulty (Experiment 3). In Experiment 4 we aimed to extend the findings from Experiments 1–3 to set sizes above 2. We controlled for increase in attention load rather than working memory load in the visual search task in Experiment 5. Finally, in Experiment 6, we used an auditory task of varied difficulty performed in the maintenance period to examine whether an attention-demanding auditory task would have an effect on feature binding of items maintained in visual working memory.

## Experiment 1

First, we investigated whether systematic increase in attentional load of a visual search task during working memory maintenance influences memory resolution. Specifically, we tested whether this manipulation results in an increase in the proportion of misbinding errors, in this case for colour and direction of motion.

### Method

#### Participants

Twelve healthy individuals (5 male) with an average age 25 years (range: 20–35), recruited from University College London participant pool, participated in this experiment. All had normal or corrected to normal vision and reported normal colour vision. They provided written consent to the procedure of the experiment, which was approved by the local ethics committee.

#### Stimuli and procedure

**Figure 1.**
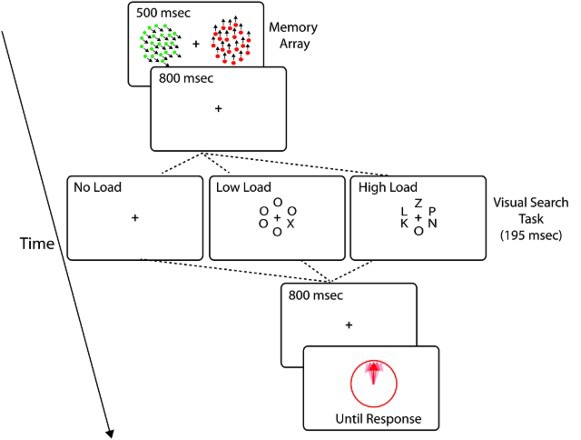
A shcematic representation of a sample trial Experiment 1. Two coloured RDKs, moving coherently in different direction of motion were presented simultaneously. In 1/3 of the trials, probe display was presented after a long retention period. In the remaining 2/3 of the trials, a visual search task of varied difficulty (low vs. high load) was presented before the presentation of the probe display. Participants were asked to respond as fast as they can and as accurately as they can to the visual search task and were asked to adjust the orientation of the response stimuli to the direction of motion of the RDK with similar colour.

A schematic representation of a trial of our dual-task design is presented in Figure 1. The general design consisted of participants being asked to hold in memory two different moving stimuli and during the delay perform an attentionally demanding task (high load/low load compared to no load) before being probed on the memory array. Participants were asked to perform both memory and attention tasks to the best of their abilities.

Each trial started with a fixation cross (500 ms) followed by the presentation of two random dot kinematograms (RDKs) on either side of fixation cross (10° of visual angle away from the centre) for 500 ms. Each RDK consisted of dots of a single colour, presented on a grey background on either side of the fixation cross. RDKs were presented in a randomly selected colour from a selection of five easily distinguishable colours (white, red, green, blue, and yellow). Participants were asked to hold in memory the directions of motion of these coloured RDKs (memory array).

Each RDK consisted of 50 dots, each covering 0.1° of visual angle. Dots were displayed within an invisible circular aperture of 150 pixels in diameter (5.7° of visual angle). Dot lifetime was 500 ms, equal to the presentation duration of RDKs. Dots reaching the edge of the circular aperture were repositioned randomly on the other side of the aperture; therefore the averaged dot density was kept constant throughout the presentation. Motion was 100% coherent (constant speed of 4.5 degree/s for all dots). Motion direction was chosen randomly from a value between 0–360° for both RDKs.

In two thirds of the trials, following a blank interval (800 ms), a visual search array was presented briefly for 195 ms, and participants were asked to perform a letter search task. They were required to press X and Z keys on the keyboard if they detected the letter X or Z, respectively, across both high and low load conditions.

In low and high load conditions, the search array consisted of an array of six letters, each positioned on a virtual circle at a radius of 2.5 cm from the centre of the screen. The letters were one of the two target letters (Z or X) and five nontargets or distractors (five Os in low load condition and letters R, K, V, S, L in high load condition). Different distractor letters made discrimination more difficult in the high load condition. Letters were presented in Ariel font and were 40 points in size (each subtending 0.6 by 0.4°). In 50% of the trials the target letter was letter “Z”, and in the remaining trials the target letter was “X”. Participants were asked to respond as fast and as accurately as they could. Auditory feedback (correct or incorrect) was provided on performance in this task. Separation between the two RDK motion directions was 86° on average (*SD* = 9) for low load trials and 91° (*SD* = 13) for high load trials. Following the visual search task, a blank interval was presented for 800 ms before the presentation of the working memory probe.

In the remaining third of trials, no visual search task was presented (Figure 1, no load condition). Separation between the two RDKs was 87° on average (*SD* = 10) in this condition. Following the presentation of the two RDKs, a blank interval of 1795 ms was displayed before the presentation of the probe display. All trial types were randomly interleaved within a block.

The probe stimulus consisted of a circle (5.7° of visual angle in diameter) presented at the centre of the screen with an arrow positioned at a random orientation—drawn from the uniform distribution (0–360°)—within the circle. The probe stimulus was presented in the same colour as one of the two RDKs in the memory array. Participants were asked to adjust the orientation of the arrow, using a mouse, to match the direction of motion of the RDK presented in the same colour in the memory array. Participants then had to press the left mouse key to confirm their response. The probability of probing any of the RDKs was kept constant for each RDK. The probe display was presented until response. Participants were informed to give equal weight to both the working memory and the visual search task in each trial.

Stimuli were generated by Cogent toolbox (www.vislab.ucl.ac.uk/Cogent/) for MATLAB and were displayed on a 21-inch CRT monitor (refresh rate: 60 Hz). A chin rest, positioned at 60 cm from the screen, ensured a 60-cm distance from the monitor.

Participants completed three blocks of 60 randomly intermixed trials per visual search condition (i.e., no, low, and high conditions) in a dimly illuminated room. Prior to the start of the experiment, participants were acquainted with the experimental apparatus and conditions by a gradual increase in the complexity of the practice trials.

#### Analysis

In each trial, both reaction times (RTs) and accuracy in the visual search task under both load conditions were calculated. In addition, overall performance in the working memory task was computed for each attention load condition as 1/standard deviation of error in recall, labelled precision. The parameter space for motion direction is circular; therefore we used Fisher's definition of standard deviation for circular data (Fisher, 1993), subtracting this value form the expected value for chance. Therefore zero precision corresponds to chance-level precision.

**Figure 2.**
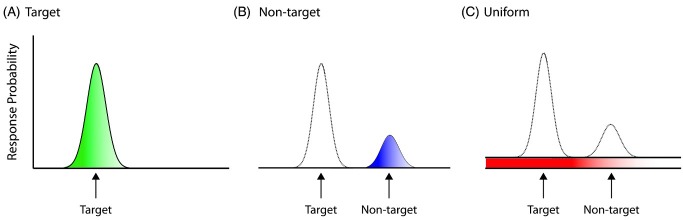
Three sources of error in memory used for modeling performance. (A) A Von Mises (circular Gaussian) distribution with concentration parameter κ, centred on the target value, capturing variability in memory for target, with the area under the distribution (shaded) being proportional to the probability of responding to the target; (B) Von Mises distribution with concentration parameter κ, centred on one of the non-target value, resulting from errors in identifying which target value belonged with the target colour (misbinding). The area under the distribution corresponds to the proportion of non-target responses and (C) A uniform distribution of error corresponding to random error, with the area under this distribution corresponding to the proportion of random responses.

Overall performance (defined here as precision of recall) does not provide information about the sources of error. In order to distinguish between these, we applied a probabilistic model proposed previously by Bays et al. (2009). This allows us to isolate the sources of error affected by loading attention resources. According to this model, there are three sources of errors in recall:

1. A Von Mises (circular Gaussian) distribution in memory centred on the target value (Figure 2A).
2. A Von Mises distribution in memory centred on nontarget value—that is, around the direction of motion of dots in the RDK that was not probed (Figure 2B).
3. A uniform distribution of error corresponding to random error or guessing (Figure 2C).

The model is described by the following equation:

$$\mu (\sigma )=\alpha \phi_{\kappa }(\theta -0)+\beta \frac{1}{m}\sum_{i}^{m}\phi_{\kappa }(\sigma -\phi_{i})+\gamma \frac{1}{2\pi }$$

Where θ corresponds to the target direction, σ is the response direction, and $\phi_{\kappa }$ is the Von Mises (circular Gaussian) distribution with a mean of zero and concentration parameter κ. The concentration parameter κ corresponds to the variability of recall of the target computed by the model, where greater κ corresponds to lower variability in the distribution. (Note that we use the term “precision” here as a measure of an individual's precision of recall. This provides no information regarding the exact sources of error. The model estimate for variability around the target item, labelled as concentration parameter κ, provides an index of one source of error contributing to overall behavioural precision.) The probability of responding with the target direction is given by α [also referred to as *p*(T)].

Successful performance in tasks similar to the one here requires memory for correct combination of motion direction and colour. Therefore error can arise as a result of incorrect conjunction of colour and motion direction (misbinding): trials where participants make an error centred on the nontarget; β corresponds to the probability of responding with such misbinding errors [also referred to as *p*(NT), or probability of making nontarget errors]. $\left\{\phi_{1},\phi_{2},\ldots \phi_{m}\right\}$ correspond to motion directions of *m* nontarget items (here *m* = 1). Probability of responding at random (γ) is calculated as 1 – α − β and corresponds to the proportion of trials where participants were guessing [also referred to as *p*(U)]. Maximum likelihood parameters of α, β, γ, and κ were obtained using expectation maximization (Myung, 2003) for each participant and under each search condition (full details in Bays et al., 2009).

However, it is important to note that model comparison between different model estimates is not possible to conduct in a straightforward manner. Hence a null effect of attentional load on one parameter and not on another does not necessarily mean that there is a difference between the effects of attention on the two model parameters (Gelman & Stern, 2006).

### Results and discussion

#### Effect of load manipulation on visual search performance

High load visual search task resulted in significantly longer mean RT and decrease in mean accuracy compared to the low load condition (744 vs. 596 ms and 83% vs. 94%), *t*(11) = 6.168, *p* < .001, for accuracy, and *t*(11) = 9.368, *p* < .001, for RT. This confirmed that attentional load was successfully manipulated in the visual search task. Trials with an incorrect response in the visual search task were excluded from analyses of the working memory task performance.

#### Visual working memory performance

The key question we wished to address is whether increasing attention load in the search task would affect memory for items already stored in working memory. Precision of working memory representations—that is, 1/distribution of error in response (see Analysis section)—was affected by visual search load manipulations [main effect of search load, *F*(2, 22) = 5.63, *p* = .011]. There was a significant decrease in the fidelity of memory representations under high load compared to no load, *t*(11) = 3.688, *p* = .004, and low load conditions [*t*(11) = 2.454, *p* = .032; *ns* after Bonferroni correction]. There was no significant difference in performance between no load and low load search conditions, *t*(11) = 0.949, *p* > .05. Thus, overall working memory performance is influenced by visual search load/difficulty.

**Figure 3.**
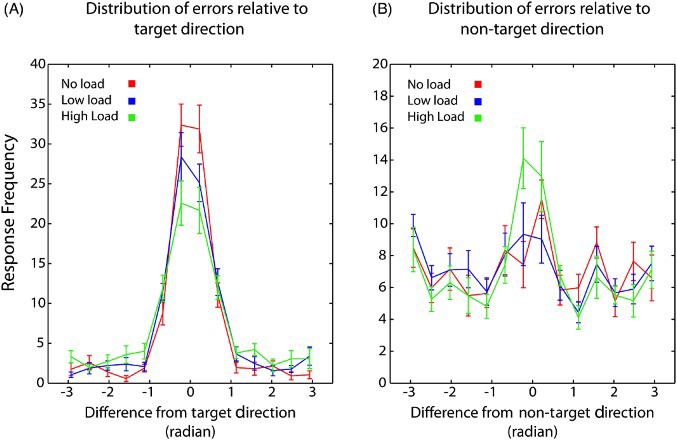
Distribution of errors relative to target and non-target motion direction, Experiment 1. (A) Frequency of response as a function of the difference between the response and the target motion direction. Under high load condition, the variability in recall of the target direction (width of the distribution) increase and the peak of the distribution centred around target value (zero) decreases. (B) Frequency of response as a function of the difference between the response and the non-target motion direction. There is a larger proportion of responses around the non-target direction under high load condition compared to the other two conditions. Error bars indicate SEM (*N* = 12).

However, the overall performance does not inform us on the sources of error that are affected by visual search load—that is, whether decrease in working memory performance was due to increase variability in response for the target feature (κ), changes in proportion of target responses [*p*(T)], proportion of nontarget responses [*p*(NT)], or increased random responses [*p*(U)]. Thus, we next examined the distribution of errors in relation to the target direction under different conditions (i.e., no, low, and high load conditions). As illustrated in Figure 3A, the proportion of responses falling close to the target direction decreased systematically as the attentional load increased. This is illustrated as a decrease in the peak of response distribution around zero—that is, target value under high load condition. Furthermore, the longer tails of the distribution under high load condition provide evidence for additional source of error (either guessing or misbinding errors) that may occur in this condition (Figure 3A). Importantly, the proportion of responses centred on the nontarget motion direction increased in the high load condition (Figure 3B). Therefore, under high load conditions, increased errors arose as a result of responding to the nontarget direction. In other words, participants erroneously misbound the colour of the probed RDK (target) with the direction of motion of the RDK that was not probed (nontarget). This is clearly illustrated in an increase in the peak of response distributions around the nontarget value under high attention load.

To quantify the possible sources of error in memory affected by visual search load, we applied a three-component model of response error to our data (see Analysis section). Maximum likelihood estimates of the probability of responding at random, the probability of responding with the target and nontarget motion direction, and variability (concentration parameter, κ) in recall of target direction were estimated. Subsequent to model fitting, outlier values were excluded. Model estimates for κ and random responses for one participant were 2.5 standard deviations above the mean values, and the probability of target responses for the same participant was 2.5 standard deviations below the mean value. Therefore for the remaining analysis, this participant was excluded.

**Figure 4.**
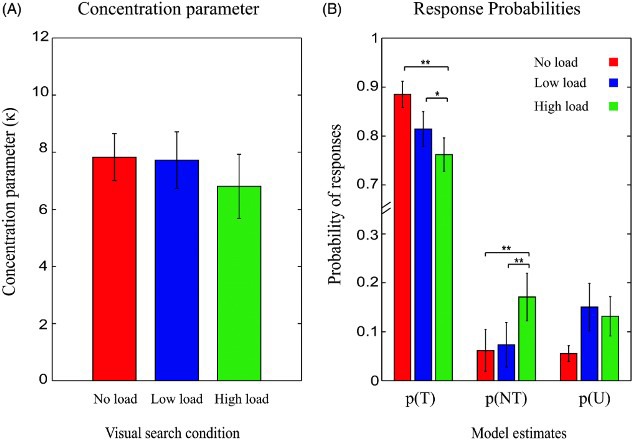
Model estimates for different sources of error in the visual working memory task for different search conditions, Experiment 1. (A) Concentration parameter did not differ significantly between different visual search conditions. (B) Probability of target responses (p(T)) decreased significantly under visual search conditions compared to no search condition. Probability of target responses (p(NT) increased significantly under high load condition compared to no load and low load conditions and probability of random responses (p(U)) did not differ significantly between different conditions. Error bars indicate SEM (*p < .05, **p < .01).

There was no significant difference in κ (i.e., variability in performance for the target motion direction; Figure 4A). Proportion of target responses varied significantly under different load conditions, *F*(2, 30) = 3.61, *p* < .039. There was a significant decrease in proportion of target responses under high load compared to no load, *t*(10) = 4.9, *p* < .001, and low load conditions [*t*(10) = 2.291, *p* = .045, *ns* after Bonferroni correction]. Further there was a marginally significant decrease in proportion of target responses under low load compared to no load condition, *t*(10) = 2.303, *p* = .052. The modulation in proportion of target responses under different load conditions was accompanied by changes in the proportion of nontarget responses. There was a significant increase in proportion of nontarget responses under high load condition compared to no load, *t*(10) = 6.5, *p* < .001, and low load conditions, *t*(10) = 3.5, *p* = .006 (Figure 4B). There was no significant difference in proportion of random responses under different search conditions (Figure 4B). The same pattern of results was obtained when all trials, regardless of search accuracy, were included in the analysis.

The results from Experiment 1 demonstrate an increase in the proportion of responses centred on the nontarget motion direction in trials where the visual search load/difficulty of the secondary task was high without any change in variability of recall for the target motion direction. Therefore, loading visual attention (by a secondary visual search task) during working memory maintenance for motion directions specifically results in feature-binding failures for items maintained in memory.

## Experiment 2

In order to establish the generality of the relationship between attention and feature binding in working memory, we aimed to extend these findings to another visual feature. In Experiment 2, we applied similar methodology to a visual working memory task but now for remembering the orientation of coloured bars, rather than remembering the direction of movement.

### Method

#### Participants

Fifteen healthy individuals (7 male) with an average age of 25 years (age range: 18–37 years), recruited from University College London participant pool, participated in this experiment. All participants reported normal colour vision and had normal or corrected-to-normal vision.

#### Stimuli and procedure

Stimuli and procedure in this experiment were similar to those in Experiment 1, except for the following changes. In each trial, two coloured oriented bars (2° × 0.3° of visual angle) were presented 10° of visual angle away from the centre on either side of fixation point for 500-ms (memory array). The colours of the bars were chosen randomly from a selection of five easily distinguishable colours (similar to those in Experiment 1). The angles of the two bars were chosen randomly from a value between 0–180°. Participants were asked to hold in memory the orientation of the two coloured bars.

Following an 800-ms delay, in two thirds of trials a visual search task with varied difficulty (low or high load condition) was presented for 195 ms. Participants were asked to perform a letter search task under low and high load conditions (similar to that in Experiment 1). Participants were asked to respond as fast and as accurately as they could in the visual search task. Separation between the two oriented bars was 44° on average (*SD* = 4) for low load trials and 45° (*SD* = 6) for high load trials. In the remaining third of the trials, after the presentation of the memory array a delay interval (1795 ms) was displayed before the presentation of the probe display (separation between oriented bars: mean = 46°, *SD* = 7).

The probe stimulus consisted of an oriented bar (2° × 0.3° of visual angle) at the centre of the screen, presented at a random orientation drawn from a uniform distribution (0–180°). The probe bar was displayed in the same colour as one of the oriented bars in the memory array. Participants were asked to adjust the orientation of the probe bar, using a mouse, to match the orientation of bar presented in the same colour in the memory array. The probability of probing any of the oriented bars was kept constant for both bars. The probe was displayed until response.

Participants completed three blocks of 60 randomly intermixed trials per visual search task difficulty (i.e., no load, low load, and high load conditions) in a dimly illuminated room. Reaction times (RTs) and accuracy on the visual search task and accuracy in the working memory task were calculated. Prior to the start of the experiment, participants were acquainted with the experimental apparatus and condition by a gradual increase in the complexity of the practice trials.

### Results and discussion

#### Effect of load manipulation on visual search performance

We replicated the findings in Experiment 1 on the effects of load on RT and accuracy in the visual search task. High load visual search task resulted in significantly longer RT and decrease in accuracy compared to low load search (548 ms vs. 449 ms and 78% vs. 92%), *t*(14) = 4.463, *p* = .001, and *t*(14) = 9.307, *p* < .001, respectively. Trials with an incorrect response in the visual search task were excluded from analyses of the working memory task performance.

#### Visual working memory performance

Overall performance in the working memory was significantly affected by the visual search condition, *F*(2, 42) = 7.028, *p* = .002. Pairwise comparison between the three visual search conditions showed that precision of performance was significantly lower under high load than under no load, *t*(14) = 6.515, *p* < .001, and low load conditions, *t*(14) = 4.535, *p* = .001, replicating the findings from Experiment 1. There was also a significant decrease in performance under low load condition compared to no load condition, *t*(14) = 4.11, *p* = .001.

We then applied the three-component model of response error to our data, and maximum likelihood estimates of κ and proportion of target, nontarget, and random responses were calculated. Table 1 shows the model estimates under different visual search load conditions.

**Table 1.** Model estimates under different visual search conditions in Experiment 2

Load	κ	*p*(T)	*p*(NT)	*p*(U)
No load	4.54 (1.7)	.89 (.14)	.03 (.05)	.08 (.1)
Low load	3.55 (1.4)	.83 (.18)	.07 (.15)	.10 (.14)
High load	3.6 (1.8)	.72 (.19)	.13 (.15)	.14 (.17)

*Note:* Means. Standard deviations in parentheses.

There was a significant effect of search condition on proportion of target responses, main effect of search condition, *F*(2, 42) = 3.46, *p* < .041. Pairwise comparison under different load conditions revealed a significant decrease in proportion of target responses under low load compared to no load condition [*t*(14) = 2.505, *p* = .025, *ns* after Bonferroni correction] and under high load compared to no load condition, *t*(14) = 5.683, *p* < .001. This was accompanied by a significant modulation in the proportion of nontarget responses. There was an increase in proportion of nontarget responses under high load condition compared to no load, *t*(14) = 3.23, *p* = .006, and low load condition, *t*(14) = 2.873, *p* = .012. There was no effect of visual search difficulty on proportion of random responses, *F*(2, 42) = 1.8, *p* > .1, and κ, *F*(2, 42) = 0.9, *p* > .3. The same pattern of results was obtained when all trials, regardless of search accuracy, were included in the analysis.

The results from Experiment 2 replicate the findings of Experiment 1; increasing the visual search difficulty resulted in an increase in proportion of binding failures with no change in variability of recall for the target orientation (i.e., no change in κ). Therefore, across a variety of visual features (colour-motion and colour-orientation) these findings suggest that attention plays a crucial role in maintenance of bound representations in working memory. Increasing the load of visual attention results in failures in binding for features of objects maintained in memory.

## Experiment 3

Binding failures in high attentional load conditions might be due to the specific role of attention in maintenance of feature-bound objects. Alternatively, they might occur because search difficulty is not high enough to influence other sources of error—that is, variability in target feature. Therefore, in Experiment 3, we increased the load of the visual search task even further (by adding more distractor letters in one of the visual search conditions, which we have termed “hyper load”) to investigate whether we can further increase binding failures or alternatively influence other sources of error.

### Method

#### Participants

Thirteen healthy individuals (6 male) with an average age of 24 years (age range: 18–29 years), recruited from University College London participant pool, with normal or corrected-to-normal vision, participated in this experiment. All participants reported normal colour vision.

#### Stimuli and procedure

Stimuli and procedure in this experiment was identical to those in Experiment 1 except for the following changes. In three quarters of the trials, after the presentation of the memory array, a visual search task was presented. The visual search displays in the low and high load conditions were identical to those in Experiment 1. A third load condition was also included in this experiment: the hyper load condition. In this condition, the search array consisted of two circular arrays (5 cm and 6.5 cm in diameter) of 6 letters each: 11 distractor letters (R, K, V, S, L, W, N, P, Y, F, J) and 1 target letter (either X or Z). The visual search array was presented for 195 ms. Participants were asked to perform a letter search task and press X or Z keys of the keyboard when detecting the letters X or Z respectably amongst the search array. Participants were asked to respond as fast and as accurately as they could. Auditory feedback was provided on performance in this task.

Identical to Experiments 1 and 2, the visual search task was followed by an 800-ms delay and the probe display. In a quarter of the trials, no visual search task was presented, and the memory array was followed by a 1795-ms delay before the presentation of the probe. Separation between the two RDK motion directions was 89° on average (*SD* = 6) for no load trials, 87° (*SD* = 7) for low load trials, 91° (*SD* = 7) for high load trials, and 87° (*SD* = 8) for hyper load trials.

### Results and discussion

#### Effect of load manipulation on visual search performance

Accuracy of search was significantly higher in the low load condition than in high load (96% vs. 90%), *t*(12) = 3.354, *p* = .01, and hyper load conditions (96% vs. 70%), *t*(12) = 9.264, *p* < .001. Furthermore, there was a significant decrease in accuracy under hyper load condition compared to high load condition, *t*(12) = 7.832, *p* < .001.

RTs were significantly faster in the low load condition than in high load (555 ms vs. 725 ms), *t*(12) = 8.387, *p* < .001, and hyper load conditions (555 ms vs. 830 ms), *t*(12) = 11.241, *p* < .001. Moreover, RTs were faster in the high load condition than in the hyper load condition, *t*(12) = 4.739, *p* < .001. The findings from the hyper load condition confirm that the load manipulation in this new condition was successful. For the rest of the analysis on performance in the working memory task, trials with incorrect responses in the visual search task were excluded from the analysis.

#### Visual working memory performance

We first investigated the behavioural consequences of increasing visual load difficulty on working memory performance. There was a significant effect of visual load on memory performance, *F*(3, 48) = 4.624, *p* < .01. There was a significant decrease in overall memory precision, compared to no load condition, in the low, *t*(12) = 2.951, *p* = .012, high, *t*(12) = 4.546, *p* < .002, and hyper load, *t*(12) = 6.017, *p* < .001, conditions, replicating and extending the findings from Experiment 1. Performance was significantly worse under hyper load condition than under low load condition, *t*(12) = 3.254, *p* < .01.

**Figure 5.**
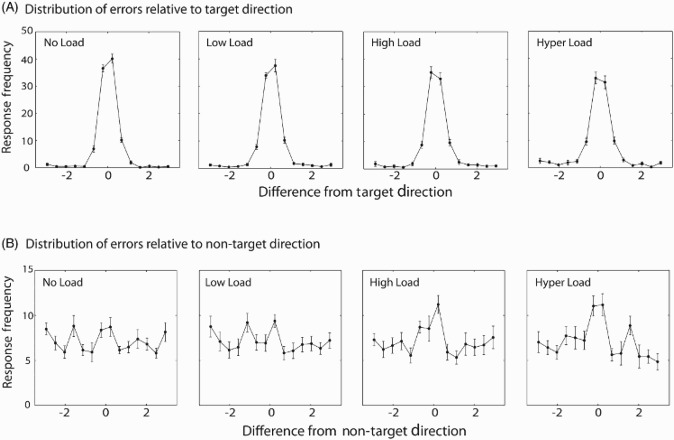
Distribution of errors relative to target and non-target directions in Experiment 3. (A) Frequency of response as a function of the difference between the response and the target direction. As visual search difficulty increased, the variability in recall (width of the distribution) around the target direction increased and the peak of distribution decreased. (B) Frequency of responses as a function of the difference between the response and the non-target direction. There is an increase in proportion of non-target responses as visual search difficulty increased from no load to hyper load conditions. Error bars indicate SEM.

Changes in overall performance were accompanied by changes in distribution of responses around the target direction under different visual search conditions, replicating and extending the findings from Experiment 1. The results illustrate that as the difficulty of the visual search increases, from no load to hyper load, the peak of responses in the working memory task around the target direction decreased. This was accompanied by an increase in the width of the distribution and the proportion of responses away from the target direction as search difficulty increased (Figure 5A).

The proportion of responses around the nontarget motion direction increased as search difficulty increased (Figure 5B), illustrating that in trials where the visual search condition was more difficult, participants were more likely to respond to the nontarget motion direction.

We further applied the three-component model of response error to our data, and maximum likelihood estimates of κ and the proportion of target, nontarget and random responses were calculated. Table 2 shows the model estimates under different visual search load conditions.

**Table 2.** Model estimates under different visual search conditions in Experiment 3

Load	κ	*p*(T)	*p*(NT)	*p*(U)
No load	10.2 (3.3)	.93 (.06)	.014 (.02)	.06 (.05)
Low load	10.6 (3.6)	.85 (.12)	.013 (.02)	.13 (.12)
High load	9.1 (4.6)	.82 (.1)	.07 (.07)	.10 (.09)
Hyper load	9.2 (5.1)	.79 (.14)	.09 (.07)	.11 (.11)

*Note:* Means. Standard deviations in parentheses.

**Figure 6.**
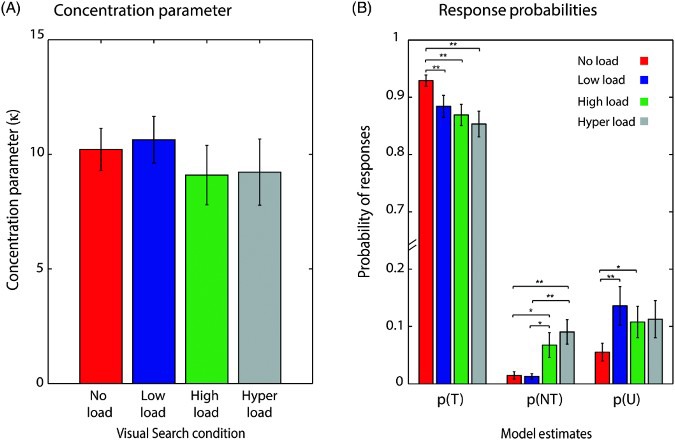
Model estimates for different sources of error in the visual working memory task for different search conditions in Experiment 3. (A) Concentration parameter did not differ significantly between different visual search conditions. (B) Probability of target responses (p(T)) decreased significantly under visual search conditions compared to no search condition. Probability of target responses (p(NT) increased significantly under high and hyper load condition compared to no load and low load conditions and probability of random responses (p(U)) increased significantly under low and high load conditions compared to no load condition only. Error bars indicate SEM (*p < .05, **p < .01).

There was no significant difference in κ -variability in memory representation for the target motion direction, *F* (3, 48) = .401, *p* > .5 (Figure 6A). Critically, there was an effect of load condition on proportion of target responses, one-way analysis of variance (ANOVA), *F*(3, 48) = 3.252, *p* < .05. Probability of target responses, as compared to no load condition, decreased significantly in the low, *t*(12) = 3.146, *p* = .008, high, *t*(12) = 4.297, *p* < .002, and hyper load, *t*(12) = 4.38, *p* < .002, conditions. The changes in probability of target responses were accompanied by changes in the proportion of nontarget responses under different load conditions, *F*(3, 48) = 6.097, *p* = .001 (Figure 6B). There was a significant increase in nontarget responses in high load condition compared to no load condition [*t*(12) = 2.55, *p* = .025, *ns* after Bonferroni correction] and low load condition [*t*(12) = 2.452, *p* = .03, *ns* after Bonferroni correction]. Importantly, there was a significant increase in nontarget responses in the hyper load condition compared to no load, *t*(12) = 3.718, *p* = .003, and low load conditions, *t*(12) = 4.224, *p* = .001. There was no significant difference in random responses, *F*(3, 48) = 1.465, *p* > .2, under different visual search conditions. The same pattern of results was obtained when all trials, regardless of search accuracy, were included in the analysis.

The findings show a systematic increase in failures to maintain bound features in working memory alone as the attentional load of the secondary task increases, replicating and extending the findings from Experiments 1 and 2. Although the estimated proportion of random responses increased in trials where the visual search task was present, it is important to note that this increase was not systematically modulated by attentional load.

## Experiment 4

In Experiments 1–3, number of items maintained in memory were below the item limit previously suggested (e.g., Anderson et al., 2011; Luck & Vogel, 1997; Luria & Vogel, 2011). Loading attentional resources by a demanding search task resulted in systematic corruption of memory for target item by the nontarget item without any change in the resolution (κ) of remembered features or complete loss of information (i.e., increase in proportion of random responses). Previous studies have used working memory set sizes of 3 or 4 items (e.g., Allen et al., 2006, 2012; Brown & Brockmole, 2010; Fougnie & Marois, 2009). Thus, in Experiment 4 we aimed to extend the findings to memory set sizes above two to examine the effects of attentional load at these set sizes.

### Method

#### Participants

Twenty healthy individuals (11 male) with an average age of 25.4 years (age range: 19–35 years), recruited from University College London participant pool, participated in this experiment. All participants reported normal colour vision and had normal or corrected-to-normal vision.

#### Stimuli and procedure

In each trial, an array of 1 to 4 oriented bars was presented for 500 ms on a grey background, followed by blank display for 800 ms. The centre of each bar was displayed on an invisible aperture 8° of visual angle away from the centre of the screen. The distance between the bars in the memory array was fixed at 180° in trials with two bars, 120° for three bars, and 90° for four bars. The position of the bars was otherwise chosen at random. The orientation of each bar in the memory array was chosen randomly from 0–180° with no minimum separation in bars presented in a trial.

Next, similar to Experiments 1 and 2, a visual search array of low or high perceptual load was presented for 195 ms, and participants were required to respond as fast and as accurately as possible to the target item (either letter Z or letter X) in the visual search display. The search array was followed by an 800-ms blank interval before the presentation of the probe display (similar to that described in Experiment 2). Prior to the start of the experiment, participants were acquainted with the experimental apparatus and conditions by a gradual increase in the complexity of the practice trials.

### Results and discussion

#### Effect of load manipulation on visual search performance

We replicated the findings in Experiments 1 and 2 on the effects of visual search load on RT and accuracy in the visual search task. Table 3 shows the average RT and accuracy in the search conditions, for different working memory set sizes. There was a main effect of search load on RT, *F*(1, 19) = 337, *p* < .0001, regardless of the number of items held in working memory [main effect of working memory load: *F*(1, 57) = 1.35, *p* = .27]. Similarly, accuracy was significantly higher for low load visual search trials than for high load visual search [main effect of search load: *F*(1, 19) = 131, *p* < .0001], regardless of working memory set size [main effect of working memory load: *F*(1, 57) = 1.12, *p* = .35]. Trials with an incorrect response in the visual search task were excluded from analyses of the working memory task performance.

**Table 3.** RT and accuracy means for low and high visual search conditions for different working memory set sizes in Experiment 4

Working memory set size	RT		Accuracy	
	Low	High	Low	High
1	590	703	91	75
2	580	698	93	76
3	585	701	90	76
4	585	705	91	75

*Note:* RT = reaction time (in ms). Accuracy in percentages.

#### Visual working memory performance

Overall working memory performance, as demonstrated in previous studies (e.g., Bays et al., 2009; Gorgoraptis, Catalao, Bays, & Husain, 2011; Zokaei, Gorgoraptis, Bahrami, Bays, & Husain, 2011), decreased as the number of items in memory increased [main effect of working memory set size: *F*(2, 38) = 11.1, *p* < .001]. For set size 1, there was no effect of visual search load on working memory precision, *t*(19) = 0.3. *p* = .8. However, for set sizes above 1, memory performance was influenced by the load of the visual search task [main effect of search load: *F*(1, 19) = 5.6, *p* = .029].

We then applied the three-component model of response error to our data, and maximum likelihood estimates of κ and the proportion of target, nontarget, and random responses were calculated. One participant was excluded from the remaining analysis since the estimated κ for this participant was 2.5 standard deviations above the mean values. Table 4 shows the model estimates for different working memory set sizes under different visual search load conditions.

**Table 4.** Model estimates under different visual search conditions for different working memory set sizes in Experiment 4

Model parameters		Memory set size			
	Search load	1	2	3	4
κ	Low	20.9 (2)	12.9 (1.4)	11.5 (1.6)	10.5 (2.4)
	High	18.8 (2.5)	12.7 (1.7)	9.3 (1.4)	9.2 (1.7)
*p*(T)	Low	.89 (.02)	.78 (.03)	.68 (.04)	.57 (.05)
	High	.90 (.2)	.70 (.04)	.61 (.04)	.49 (.03)
*p*(NT)	Low	0	.10 (.02)	.27 (.04)	.40 (.09)
	High	0	.17 (.04)	.28 (.05)	.47 (.1)
*p*(U)	Low	.10 (.3)	.10 (.02)	.05 (.01)	.03 (.008)
	High	.10 (.02)	.12 (.02)	.07 (.02)	.03 (.01)

*Note:* Means. Standard deviations in parentheses.

For memory set size of 1, there was no effect of search load on any of the model estimates, presumably because no other items were maintained to corrupt the memory for target. For the remaining analysis, data from set sizes 2, 3, and 4 were used. Proportion of target responses decreased significantly as the memory set size increased [main effect of memory set size: *F*(2, 36) = 43.6, *p* < .001]. Moreover, there was a significant effect of search load on proportion of target responses [main effect of search load: *F*(1, 18) = 17.6, *p* < .01], with the proportion of target responses decreasing significantly in high compared to low load search condition in memory set sizes of 2, *t*(18) = 2. 3, *p* = .03, and 4, *t*(18) = 2.2, *p* = .04, and marginally significant for set size 3, *t*(18) = 2.01, *p* = .054, *ns* after Bonferroni correction.

Proportion of responses centred on one of the nontargets increased as number of items increased (main effect of memory set size; *F*(2, 36) = 91, *p* < .001) but was also modulated by search difficulty [main effect of search load: *F*(1, 18) = 10, *p* < .01]: Nontarget responses were higher in high than in low load search conditions.

Proportion of nontarget responses increased significantly in high compared to low load condition for memory set size 2, *t*(18) = 2.2, *p* = .046.

κ decreased significantly as memory set size increased [main effect of memory set size: *F*(2, 36) = 5.9, *p* = .006] and was also modulated by search difficulty [main effect of search load: *F*(1, 18) = 4.6, *p* = .045]. Variability in memory for the target orientation (κ) decreased significantly in high compared to low search load in memory set size 3 [*t*(18) = 2.8, *p* = .01, *ns* after Bonferroni correction]. There was no significant change in κ for set size 2, *t*(18) = 0.16, *p* > .8, and 4, *t*(18) = 0.1.2, *p* > .2, for high versus low search difficulty. Proportion of random responses decreased with an increase in memory size [main effect of memory set size: *F*(2, 36) = 13.8, *p* < .001] but was not modulated by search difficulty [main effect of search load: *F*(1, 18) = 2.01, *p* = .2]. The same pattern of results was obtained when all trials, regardless of search accuracy, were included in the analysis.

Therefore, similar to our findings from previous experiments, visual search task with high level of difficulty, performed during working memory maintenance, primarily impaired performance by systematically corrupting target memory by nontarget items, regardless of number of objects retained. The parameter κ was also modulated, to a lesser extent, by visual search difficulty, pointing to the conclusion that with larger set sizes, where the resolution of memory for the target item is already coarse, loading attentional resources results in noisier representations.

#### Comparing the magnitude of change in model parameters for Experiments 1–4

For Experiments 1–4, we also investigated the magnitude of change in each parameter for each participant by examining the difference between low and high visual search load conditions, normalized by the mean value of each parameter. Thus, for example, for κ we computed: κ (high-low search load)/mean κ. Analogous calculations were also performed for *p*(NT) and *p*(U). We then statistically compared the change in κ or *p*(U) directly against the change in *p*(NT). The magnitude of change in low versus high load conditions was statistically higher for *p*(NT) responses than for those in either κ [Experiment 1: *t*(10) = 5.7, *p* < .001; Experiment 2: *t*(14) = 2.25, *p* < .05; Experiment 3: *t*(12) = 3.2, *p* < .05; Experiment 4: *F*(1, 18) = 12.1] or *p*(U) [Experiment 1: *t*(10) = 2.85, *p* < .05; Experiment 3: *t*(12) = 2.44, *p* < .05], in all experiments, except only for the comparison between *p*(U) and *p*(NT) in Experiment 2. Together with the selective replication of effects across several experiments, this analysis demonstrates that the effect of load during maintenance is higher on *p*(NT) than on either κ or *p*(U).

## Experiment 5

In previous experiments, the search array, regardless of search difficulty, was presented for 195 ms. Thus, in more difficult conditions—that is, hyper load condition (Experiment 3)—search might not have been completed reliably during the time the array was visible. It might be argued that in order to perform the task accurately, participants might have needed to encode the search array in memory and then complete the search over the memory representations. Therefore, under these conditions, increase in misbinding errors could theoretically have arisen due to increase in memory load rather than attention load. In Experiment 5 we aimed to address this problem in a condition where the presentation duration of the search array in the hyper load condition was increased to ensure successful search performance while the search array was visible.

### Method

#### Participants

Twelve healthy individuals (7 male) with an average age of 29.5 years (age range: 23–38 years), recruited from University College London participant pool, participated in this experiment. All participants reported normal colour vision and had normal or corrected-to-normal vision.

#### Stimuli and procedure

Stimuli and procedure in this experiment were similar to those in Experiment 3, except for the following changes. Eight hundred ms after the presentation of the memory array (2 RDKs) a hyper load visual search task was presented. In half of the trials, the visual search array was presented for 195 ms, similar to Experiment 3, and in the remaining trials, the presentation of the search array was increased to 600 ms (50 ms per letter in the search array) to ensure reliable search while the array was visible. The search array was followed by a 2-s delay interval in the short and 1595-ms delay in the long presentation condition. Similar to previous experiments, participants were asked to perform a letter search task and to press X or Z keys of the keyboard when detecting the letters X or Z, respectively, amongst the search array. Participants were asked to respond as fast and as accurately as they could. Auditory feedback was provided on performance in this task. Separation between the two RDK motion directions was 92° on average (*SD* = 6) for no load trials, 90° (*SD* = 6) for low load trials, and 88° (*SD* = 6) for high load trials.

### Results and discussion

Accuracy in the visual search task was marginally higher in the longer than in the shorter presentation duration (71% vs. 81%, respectively), *t*(11) = 2.02, *p*= .06. RT (calculated from the onset of search display) did not differ between the two conditions (854 ms vs. 850 ms for short and long durations, respectively), although note that in the short display condition, average RT was ∼600 ms after search task had been presented.

Working memory performance, however, was unaffected by the search task duration. Overall performance and κ and proportion of target, nontarget, and random responses were not significantly different between the two conditions (*p* > .4 for all comparisons). Table 5 shows the mean values for all working memory parameters between the two search display conditions. The lack of a significant difference in performance between the two search time conditions is, of course, a null result. It is important to bear this in mind if one accepts the null hypothesis that performance was unaffected by search time.

**Table 5.** Precision and model estimates under different visual search conditions in Experiment 5

Display duration (ms)	Precision	κ	*p*(T)	*p*(NT)	*p*(U)
Short: 195	.8 (.09)	10.2 (1.2)	.78 (.03)	.12 (.02)	.10 (.03)
Long: 600	.86 (.14)	12.2 (2.5)	.75 (.04)	.145 (.03)	.10 (.04)

*Note:* Means. Standard deviations in parentheses.

Therefore, increasing the visual search times to ensure reliable detection of the target did not influence the effects of this secondary task on memory performance and proportion of responses centred on nontarget motion direction. Thus, the search times in previous experiments reported here were sufficient to load attention/executive resources without taxing/loading working memory.

## Experiment 6

In Experiments 1–4, we examined the role of visual attention in maintenance of bound features in visual working memory. In Experiment 6, we investigated whether auditory load would also result in failures of binding in visual working memory. Previously, studies have applied continuous verbal tasks to investigate the role of attention resources on working memory encoding and maintenance. When participants were asked to perform a verbal task (i.e., counting backwards in three from a three-digit number) or an auditory tone judgement task (Morey & Bieler, 2013), during the encoding or retention phase of each trial, a significant reduction in memory performance was observed (Dell'Acqua & Jolicoeur, 2000; Morey & Bieler, 2013; Morey & Cowan, 2005; Stevanovski & Jolicœur, 2007). However, these studies have not found specific impairment in memory for bound objects under continuous verbal task performance (Allen et al., 2006). In Experiment 6, we used an attention-demanding auditory task with varied difficulty that closely resembles the visual search task in previous experiments, in the maintenance period of a visual working memory task.

### Method

#### Participants

Twelve healthy native English speakers (5 female) with an average age of 26 years (range: 19–47) participated in this experiment. All participants reported normal colour vision and had normal or corrected-to-normal vision.

#### Stimuli and procedure

Stimuli and procedure in this experiment were similar to those in Experiment 1, except for the following changes. Following a blank interval of 800-ms delay, after the presentation of the memory array (2 RDKs), participants were presented with an auditory stimulus (a word) spoken by either a male or a female voice. The words were chosen from a selection of words from a previous study, specially selected to have either a negative or a positive connotation (Meteyard, Zokaei, Bahrami, & Vigliocco, 2008). For example, the word “decay” has been found to have a negative meaning associated with it, whereas the word “raise” has a positive one. The words from both groups were matched on number of letters. The maximum length of audio files was 1 s, if an audio file was shorter than this time, a blank interval was added to match the delay interval within all trials.

In a third of the trials, participants were asked to make a gender judgement on the spoken word—that is, to press a key (Z key) if the word was presented in a male's voice and to press another key (key X) if the word was presented in a female's voice. This acted as the low auditory load condition. In another third of the trials, participants were asked to make a judgement regarding the meaning of the words: They were requested to press the Z key if the word had a positive meaning and to press X if the word had negative meaning. This condition acted as the high attention load condition. It was very different from the visual search used to load attention in the previous experiments, in terms of both sensory modality and the nature of the attention task.

Participants were asked to respond as accurately and as fast as possible. Auditory feedback was provided for performance in the visual search task. In the remaining third of the trials, no audio stimuli were presented to the participants (no load task). After a blank delay of 800 ms following the presentation of the audio file, the probe display for the working memory task was presented (similar to Experiment 1). The probe displayed until response, and probability of probing any of the RDKs was kept constant for both RDKs.

Participants completed three blocks of 60 trials: one block per auditory load condition. Reaction times (RTs) and accuracy on the auditory task and accuracy in the working memory task were calculated. Prior to the start of the experiment, participants were acquainted with the experimental apparatus and different conditions by a gradual increase in the complexity of the practice trials.

### Results and discussion

#### Effect of load manipulation on auditory attention task

We first examined whether auditory attention load manipulations were successful. RTs were calculated from the onset of audio files since decision making on the gender of the speaker could be made before the word was fully spoken. The results showed that high auditory load resulted in significantly longer RTs and decrease in accuracy compared to low load condition (1500 vs. 1300 ms, 91% vs. 96%), *t*(11) = 2.549, *p* = .027, for accuracy, and *t*(12) = 2.7, *p* = .02 for RT. These results confirm that our auditory load manipulations were successful. Trials with incorrect response in the auditory task were excluded from the rest of the analysis.

#### Visual working memory performance

There was a significant decrease in precision of memory under high load condition compared to no load condition, *t*(11) = 3.454, *p* = .005, and compared to low load conditions, *t*(11) = 3.175, *p* = .009. There was no difference in performance between no load and low load conditions, *t*(11) = 0.338, *p* > .3.

Model estimates for κ and proportion of target, nontarget, and random responses are presented in Table 6. The decrease in precision of memory under high load condition was accompanied by a decrease in proportion of target responses under high load condition compared to no load condition, *t*(11) = 3.381, *p* = .006. Importantly, proportion of nontarget responses was also modulated by auditory load condition, *F*(2, 33) = 3.697, *p* = .036. There was a significant increase in proportion of nontarget responses under high load compared to no load condition, *t*(11) = 2.684, *p* = .021. A similar pattern of results was observed when comparing nontarget responses under high load and low load conditions, *t*(11) = 2.243, *p* = .046. There was no significant difference under different load conditions in either κ, *F*(2, 33) = 0.68, *p* > .5, or proportion of random responses, *F*(2, 33) = 0.554, *p* > .5. The same pattern of results was obtained when all trials, regardless of search accuracy, were included in the analysis.

**Table 6.** Model estimates under different visual search conditions in Experiment 6

Load	κ	*p*(T)	*p*(NT)	*p*(U)
No load	9.5 (1.04)	.89 (.03)	.02 (.008)	.085 (.03)
Low load	8.6 (1.04)	.83 (.07)	.03 (.02)	.13 (.05)
High load	7.89 (0.09)	.79 (.03)	.8 (.15)	.14 (.03)

*Note:* Means. Standard deviations in parentheses.

Increasing the demand of an attention-demanding auditory task during maintenance resulted in an increase in failures in correct maintenance of integrated objects in visual working memory. Thus, some of the recourses essential for maintenance of feature-bound visual objects may be shared across modalities.

## General Discussion

The present study focused on understanding the role of attention in maintenance of feature-bound objects in working memory. Despite the close connection between attention and working memory (Awh & Jonides, 2001; Chun, 2011; Chun et al., 2011; Rensink, 2000; Wheeler & Treisman, 2002), the extent of overlap between the two processes remains unclear and controversial. Here, we investigated this issue by employing a relatively new technique that allows us to decompose the types of error made when participants retrieve an item from working memory (Bays et al., 2009; Zhang & Luck, 2008).

Our findings point to a role of a resource used in attention-demanding tasks that is also important for maintaining correct conjunction of features in visual working memory. Loading visual attention resources primarily resulted in an increase in incorrect binding of visual features belonging to items already stored in working memory. Memory for conjunction of motion direction and colour (Experiments 1 and 3) and orientation and colour (Experiment 2) and for different working memory set sizes (Experiment 4) was impaired when an unrelated task with high demand was performed during memory maintenance. In these experiments, the same pattern of results was obtained when all trials, regardless of search accuracy, were included in the analysis.

Systematic loading of attention resources, whether visual (Experiments 1–5) or auditory (Experiment 6), during maintenance resulted in an increase in the frequency of binding failures—that is, the proportion of responses attributed to nontarget values. In other words, participants incorrectly bound the colour of the probed item (i.e., the target item) with either motion direction or orientation of nontarget items. Moreover, the results demonstrated above-chance performance in both working memory and the secondary task in all conditions, confirming that participants did perform both tasks.

Many authors have pointed out the close relationship between the attention and working memory (e.g., Awh & Jonides, 2001; Chun, 2011; Chun et al., 2011; Fougnie & Marois, 2006; Lavie, 2005; Rensink, 2000; Wheeler & Treisman, 2002). However, the literature provides conflicting evidence concerning automatic versus resource-demanding maintenance of bound objects in memory. Existing models invoke very different functional roles of attention in working memory processes. On the one hand, some researchers propose that the process of memory maintenance is automatic for integrated objects (Hollingworth, 2003; Johnson et al., 2008; Luck & Vogel, 1997; Luria & Vogel, 2011) while others argue for a strong case of attention—or resources shared with attention to sensory stimuli—playing a crucial role, stating that remembered objects will collapse into disintegrated features in the absence of attention (Chun, 2011; Wheeler & Treisman, 2002).

Recently it has been shown that errors in recalling the colour and orientation of an object can be uncorrelated—that is, an error can be made with respect to one feature independently of the other (Bays, Wu, & Husain, 2011). Uncorrelated errors such as these—between features belonging to one object—provide evidence for independent storage of each feature category (see also Fougnie & Alvarez, 2011, for similar findings). Hence a feature binding mechanism to maintain integrated objects in working memory may be required. The findings from the present study would suggest that maintenance of integrated objects (comprising different features) depends, at least in part, on available attention resources.

Model estimates from our study support the role of attention resources in working memory maintenance of feature-bound objects. The resolution of memory for the target feature (the model estimate of κ, width of the modelled distribution) was not influenced by the presence of a secondary task, irrespective of its difficulty, even for memory set size of 4. With regard to maintenance of bound objects, however, such resources appear to be crucial. Taxing attention resources caused an increase in misbinding—but not in random guessing—of features held in working memory. That is, there was an increase in errors arising from incorrect binding of features within objects maintained in working memory when attention resources were concurrently loaded. These findings are consistent with models that argue for a crucial role of attention in working memory maintenance for feature-bound objects (Bays et al., 2011; Chun et al., 2011; Wheeler & Treisman, 2002) and further suggests a rather selective overlap between working memory and attention processes. However, it is also important to note that finding a significant effect in *p*(NT) but no effect in *p*(U) responses under different load conditions does not necessarily mean that there is a difference between the effects of attention manipulation on these two model parameters (Gelman & Stern, 2006) since they have not been compared directly—an analysis that is not possible to conduct in a straightforward manner.

By applying dual-task designs, previous studies have provided evidence pointing to an overlap between attention and working memory processes. For example, investigations have demonstrated that loading working memory also influences visual search (e.g., Emrich et al., 2010; Oh & Kim, 2004; Woodman & Luck, 2004, but also see Woodman, Vogel, & Luck, 2001 for conflicting findings). Similarly, researchers have shown larger impairment in memory for bound objects in the presence of an attention-demanding secondary task (Brown & Brockmole, 2010; Fougnie & Marois, 2009; Stefurak & Boynton, 1986). Therefore, loading either system (i.e., attention or working memory resource) can result in impairments in the other process.

Findings using dual-task designs have, however, been inconsistent but it remains unclear why this might be. As discussed in the introduction, change-detection tasks, usually used in such dual-task designs, may be influenced by magnitude of change (Keshvari et al., 2012), as well as probe presentation and analysis techniques (Allen et al., 2012; Brown & Brockmole, 2010; Wheeler & Treisman, 2002). The discrepancy between the present results and previous work (Allen et al., 2006, 2012; Baddeley et al., 2011; Johnson et al., 2008; Yeh et al., 2005) might potentially be explained by differences in methodology. A change-detection paradigm that systematically varies the magnitude of change should theoretically yield similar findings to those of the present study, although this remains to be investigated. The secondary visual search tasks employed here are established methods of systematically loading attention resources (e.g., Lavie, 2005). However, the fact that the use of a continuous, analogue measure of working memory report has not previously been used in dual-task designs to investigate feature binding might be an important reason for differences in results.

Rather than invoking a resource shared by attention and working memory, it might be argued that the results reported here might be explained by a specialized, separate mechanism that is involved—at least in part—in both processes, but is not part of an attention or working memory module. This is certainly a logical possibility. However, considering our findings, such a mechanism would seem to be very specialized because taxing this resource results in very specific impairments, leading to errors in feature binding of objects retained in memory. Alternatively, the pattern of findings may be due to disruption of the verbal coding of memory stimuli by a highly demanding secondary task, a possibility that may be addressed by future investigations.

Taken together, the results from the present study point to an attention resource that is also essential for maintenance of bound representations in working memory. Taxing this resource while participants are asked to maintain bound representations results in a specific type of error: feature binding failures. Based on these findings, we propose a limited overlap in resources recruited in visual search and those essential primarily for maintenance of integrated features in working memory. An important challenge for future research will be to clarify the neural and psychological qualities of this resource and to determine its capacity limitations.
